# Utility of ultrasound in managing acute medical conditions in space: a scoping review

**DOI:** 10.1186/s13089-023-00349-y

**Published:** 2023-12-12

**Authors:** Parsa Asachi, Ghadi Ghanem, Jason Burton, Haig Aintablian, Alan Chiem

**Affiliations:** 1 David Geffen School of Medicine at UCLA, 855 Tiverton Dr, Los Angeles, CA 90024 USA; 2 University of California, Los Angeles Library, Los Angeles, CA USA; 3 Department of Emergency Medicine, David Geffen School of Medicine UCLA, Los Angeles, CA USA; 4 Department of Emergency Medicine, David Geffen School of Medicine UCLA, Olive View UCLA Medical Center, Los Angeles, CA USA

**Keywords:** Space, Spaceflight, Space medicine, Aerospace medicine, Microgravity, Ultrasound, Sonography, Emergencies, Diagnostic imaging

## Abstract

**Background:**

In long-distance spaceflight, the challenges of communication delays and the impracticality of rapid evacuation necessitate the management of medical emergencies by onboard physicians. Consequently, these physicians must be proficient in tools, such as ultrasound, which has proven itself a strong diagnostic imaging tool in space. Yet, there remains a notable gap in the discourse surrounding its efficacy in handling acute medical scenarios. This scoping review aims to present an updated analysis of the evidence supporting the role of ultrasound in diagnosing acute conditions within microgravity environments.

**Methods:**

A systematic search was executed across three bibliographic databases: PubMed, EMBASE (Embase.com), and the Web of Science Core Collection. We considered articles published up to February 25, 2023, that highlighted the application of ultrasound in diagnosing acute medical conditions in either microgravity or microgravity-simulated settings. Exclusions were made for review papers, abstracts, and in-vitro studies.

**Results:**

After removing duplicates, and filtering papers by pre-determined criteria, a total of 15 articles were identified that discuss the potential use of ultrasound in managing acute medical conditions in space. The publication date of these studies ranged from 1999 to 2020. A relatively similar proportion of these studies were conducted either on the International Space Station or in parabolic flight, with one performed in supine positioning to simulate weightlessness. The included studies discuss acute pathologies, such as abdominal emergencies, decompression sickness, deep venous thrombosis, acute lung pathologies, sinusitis, musculoskeletal trauma, genitourinary emergencies, and ocular emergencies.

**Conclusions:**

While ultrasound has shown promise in addressing various acute conditions, significant knowledge gaps remain, especially in gastrointestinal, cardiac, vascular, and reproductive emergencies. As we venture further into space, expanding our medical expertise becomes vital to ensure astronaut safety and mission success.

**Supplementary Information:**

The online version contains supplementary material available at 10.1186/s13089-023-00349-y.

## Introduction

As humanity reaches deeper into the cosmos, safeguarding the health of astronauts on extended missions becomes increasingly challenging. The microgravity environment in space has been shown to precipitate specific health issues, including bone demineralization, muscle atrophy, and cardiovascular deconditioning [1–3]. In addition, humans in space are still prone to the same medical conditions that are experienced on Earth, including acute emergencies that may risk a crewmember’s life, necessitating evacuation and termination of the mission. On Earth, clinicians have a wide range of diagnostic and treatment options to address these issues. However, the limited resources and dimensions of a spacecraft make it impossible to use large diagnostic machines, such as CT or MRI scanners. In this context, ultrasound has emerged as a promising solution [4]. This compact, non-invasive tool has proven to be a valuable resource for diagnosing and managing medical conditions in space.

Training astronauts in ultrasound has been a complex but necessary endeavor. Studies have shown that even with a short training, non-physician operators can become familiar with ultrasonographic findings and produce diagnostic-quality ultrasound images comparable to those obtained by emergency medicine residents [5]. With the Advanced Diagnostic Ultrasound in Microgravity (ADUM) program launched as part of the National Aeronautics and Space Administration (NASA) Expedition 8 mission in 2003, astronauts successfully gathered clear diagnostic ultrasound images on themselves and their crewmates with real-time remote assistance from mission control [6–8, 12, 25]. For missions, where real-time communication is impractical, such as those to Mars, "just-in-time" training methods have been developed, allowing astronauts to independently obtain diagnostic ultrasound images by viewing ultrasound tutorials while performing scans [8].

Despite these advancements, future missions to Mars and beyond will pose a new challenge to those on board. The 4–22 min of one-way communication delay between the two planets and 9–12 months of travel time back to Earth make remotely managing medical emergencies and conducting medical evacuations impractical and unsafe. These obstacles necessitate a need for increased medical expertise onboard and potentially requiring a new generation of physician astronauts. As a response for this need, new aerospace medicine fellowship programs are being developed across the nation. These programs seek to train future space medicine physicians to be able to directly manage acute medical emergencies on board and include a comprehensive ultrasound training curriculum. Given that these curricula are grounded in current aerospace medicine literature, it is crucial to conduct extensive research on the unique sonographic manifestations of acute diseases in microgravity environments.

The role of ultrasound in space has been the subject of past review papers [4, 9], but none are more current than 2011, and are not scoping in nature. In addition, Sargsyan et al. developed a comprehensive NASA handbook in 2006 encompassing the ultrasound capabilities of the ISS and the feasibility of specific ultrasound maneuvers that have been demonstrated either in simulated microgravity conditions or in space [10].

This scoping review aims to build on established literature and provide a focused update on the current evidence for the use of ultrasound particularly for diagnosing acute medical conditions in microgravity environments. By identifying gaps in the current knowledge, this review hopes to stimulate further research in this area and contribute to the training of future space medicine physicians, especially those who may 1-day manage acute medical conditions inflight. The ability to diagnose and manage acute medical conditions in space is not only vital for the health and safety of astronauts, but also for the success of long-duration space missions.

## Methods

### Literature search strategy

Searches were conducted on February 25, 2023, utilizing three bibliographic databases: PubMed, EMBASE (Embase.com), and the Web of Science Core Collection. Searches were constructed and then translated into the syntax and controlled vocabulary, if available, of the selected databases (Additional file 1). The search terms focused on two broad categories, ultrasonography and ultrasound and the states of weightlessness, zero gravity, and microgravity. No date limiters were used in the searches.

### Inclusion and exclusion criteria

We included all English articles published before February 25, 2023, that demonstrated the utility of ultrasound in diagnosing acute medical conditions in microgravity or microgravity-simulated environments. We excluded review papers, abstracts, and in-vitro studies. We did not restrict our search to any dates, and studies from all countries were included.

## Results

### Study selection

**Fig. 1:**
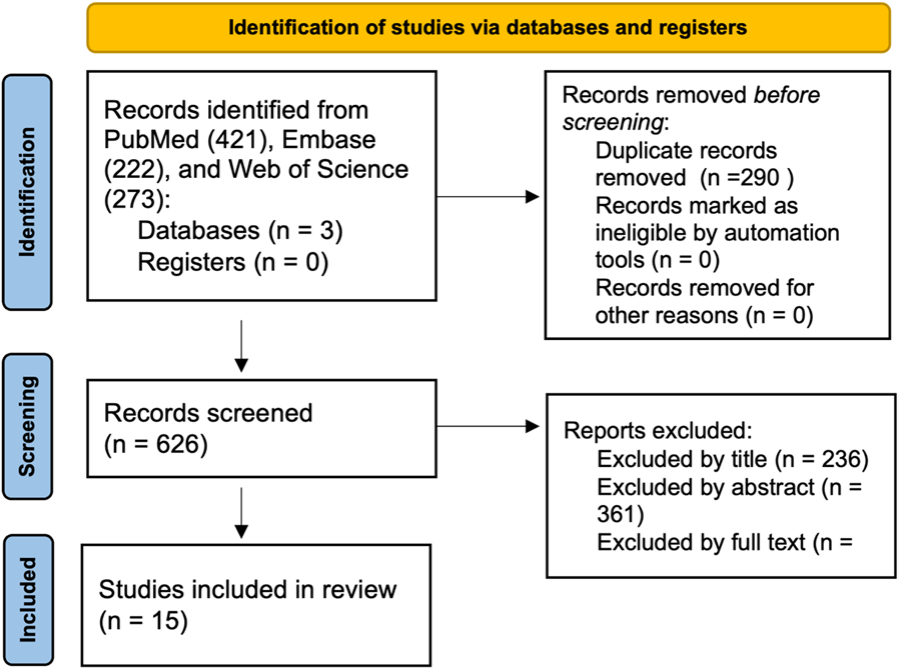
Flowchart of study selection for scoping review

In the preliminary search, PubMed yielded 421 articles, EMBASE yielded 222, and Web of Science yielded 273. Records were downloaded into Zotero (Corporation for Digital Scholarship, Vienna, VA, USA) and deduplicated using Microsoft Excel (Microsoft, Redmond, WA, USA), yielding a total of 626 articles. In the first round, two reviewers (PA and GG) independently screened the articles by title to ensure that the articles were relevant to the inclusion criteria. The reviewers were blinded from each other’s decisions, and in the event of a disagreement, a discussion was had between the two reviewers regarding the reasoning of inclusion or exclusion and a final decision was made. Deviations were found to be minimal. The articles were re-screened by abstract, then by full text using the same procedure (Fig. 1). A total of fifteen articles qualified for inclusion in this scoping review.

### Study characteristics

The fifteen studies that were selected for this scoping review dated between the years of 1999 to 2020. Out of these studies, 8 were conducted on the International Space Station, 6 were in parabolic flight simulating microgravity, and 1 was in supine to simulate weightlessness. In addition, 10 were primarily from the U.S. Three were joint studies from Canada and the U.S., one was from Sweden, and one was a joint international paper involving the U.S., Russia, and France (Table 1). The subjects of the studies encompassed a range of topics, including abdominal emergencies, decompression sickness, deep venous thrombosis, acute lung pathologies, sinusitis, musculoskeletal trauma, genitourinary crises, and ocular emergencies.

**Table 1:** Overview of the fifteen included studies, ordered by publication date, with details on the study settings: the International Space Station (ISS), parabolic flights simulating microgravity, and terrestrial human subjects positioned supine, simulating weightlessness

Paper Title	Authors	Date Published	Country	Setting
Venous thrombosis during spaceflight	Auñón-Chancellor SM, Pattarini JM, Moll S, Sargsyan A	2020	USA	ISS
Assessment of jugular venous blood flow stasis and thrombosis during spaceflight	Marshall-Goebel K, Laurie SS, Alferova IV, et al	2019	USA/France/Russia	ISS
Real‐time ultrasound assessment of astronaut spinal anatomy and disorders on the international space station	Garcia KM, Harrison MF, Sargsyan AE, Ebert D, Dulchavsky SA	2018	USA	ISS
New heights in ultrasound: first report of spinal ultrasound from the international space station	Marshburn TH, Hadfield CA, Sargsyan AE, Garcia K, Ebert D, Dulchavsky SA	2014	USA/Canada	ISS
Ultrasonographic evaluation of sinusitis during microgravity in a novel animal model	Benninger MS, McFarlin K, Hamilton DR, et al	2010	USA	Parabolic
Venous gas emboli and exhaled nitric oxide with simulated and actual extravehicular activity	Karlsson LL, Blogg SL, Lindholm P, Gennser M, Hemmingsson T, Linnarsson D	2009	Sweden	Supine
Diagnostic Ultrasound at MACH 20: Retroperitoneal and Pelvic Imaging in Space	Jones JA, Sargsyan AE, Barr YR, et al	2009	USA	ISS
Percutaneous bladder catheterization in microgravity	Jones JA, Kirkpatrick AW, Hamilton DR, et al	2007	USA	Parabolic
Ocular examination for trauma; clinical ultrasound aboard the International Space Station	Chiao L, Sharipov S, Sargsyan AE, et al	2005	USA	ISS
FAST at MACH 20: Clinical Ultrasound Aboard the International Space Station	Sargsyan AE, Hamilton DR, Jones JA, et al	2005	USA	ISS
Evaluation of Shoulder Integrity in Space: First Report of Musculoskeletal US on the International Space Station	Fincke EM, Padalka G, Lee D, et al	2005	USA	ISS
Sonographic detection of pneumothorax and hemothorax in microgravity	Hamilton DR, Sargsyan AE, Kirkpatrick AW, et al	2004	USA	Parabolic
Focused Assessment with Sonography for Trauma in Weightlessness: A Feasibility Study	Kirkpatrick AW, Hamilton DR, Nicolaou S, et al	2003	Canada/USA	Parabolic
Percutaneous aspiration of fluid for management of peritonitis in space	Kirkpatrick AW, Nicolaou S, Campbell MR, et al	2002	Canada/USA	Parabolic
Endoscopic surgery and telemedicine in microgravity: developing contingency procedures for exploratory class spaceflight	Jones JA, Johnston S, Campbell M, Miles B, Billica R	1999	USA	Parabolic

## Discussion

### Abdominal emergencies

Terrestrially, the standard of care for workup of acute traumatic injury is the focused assessment with sonography for trauma (FAST) exam, where the clinician classically uses ultrasound to scan the pericardium and three gravity-dependent intra-abdominal spaces to identify pathologic fluid. This exam has been proven to be a fast and effective method which has guided initial trauma decision making in the emergency room for over 30 years. [11] Because the FAST exam requires imaging of gravity-dependent areas of the abdomen, there is concern that microgravity may lead to a variable distribution of fluid in the abdominal cavity, potentially leading to false negatives.

**Fig. 2:**
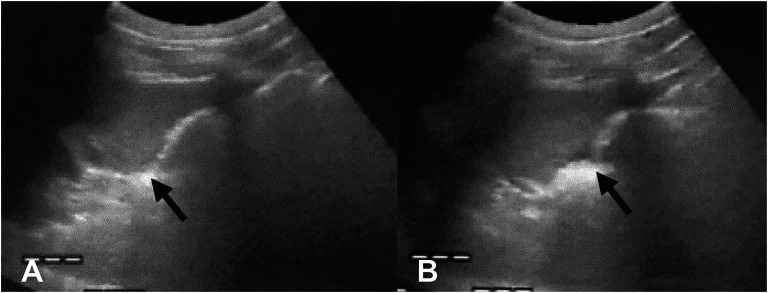
FAST exam in parabolic flight depicting intraperitoneal fluid (arrows) in Morrison’s pouch during Earth’s gravity (**A**) and microgravity (**B**). Due to surface tension, the fluid coalesced into a larger collection and was able to be better distinguished from the liver and kidney in microgravity [13]

Sargsyan et al. described the first FAST exam conducted on the international space station (ISS) by a nonphysician astronaut crewmember who was remotely guided by an experienced sonographer on Earth [12]. It was demonstrated that the crewmember could effectively perform the exam with no discernable differences between images obtained in orbit and those performed terrestrially. In a study investigating FAST exams on porcine models injected with intraperitoneal fluid during parabolic flight, results in microgravity mirrored those in normal gravity. Notably, Morrison’s pouch remained the most sensitive site for detecting intraperitoneal fluid (Fig. 2) [13]. The fluid also appeared more echogenic due to the dominating surface tension forces in microgravity. However, the study suggested that the 1.8 g exposure during parabolic flight may have centrifuged the fluid to dependent areas before 0 g imaging, potentially affecting the results [13]. Future experiments in true microgravity, using models simulating acute hemoperitoneum, could assess if standard views detect intra-abdominal fluid with equal accuracy without centrifugation effects.

Other studies have demonstrated that researchers were able to utilize ultrasound to accurately identify and aspirate intra-peritoneal fluid (which represented peritonitis) in a porcine model during weightlessness [14]. Kirkpatrick et al. also found that intra-peritoneal fluid tended to collect in the para-colic gutters during weightlessness.

### ***Decompression sickness***

Decompression sickness (DCS) is a significant health concern related to extravehicular activities (EVA) in spaceflight. The rapid transition from the spacecraft’s standard pressure to a considerably lower pressure inside the EVA suits can theoretically precipitate DCS. EVAs are integral to space missions, and their frequency is expected to rise with upcoming lunar and Martian explorations, which may put astronauts at increased risk of DCS. However, there have not been any reported cases of DCS in space thus far. While DCS is mainly a clinical diagnosis on Earth, flight surgeons may need additional diagnostic modalities to increase the confidence of their medical decision making when caring for crewmembers in spaceflight. Hugon et al. conducted a terrestrial study showcasing that ultrasound had high sensitivity in detecting the presence of venous gas emboli (VGE), a finding strongly correlated with the risk of developing DCS [15].

**Fig. 3:**
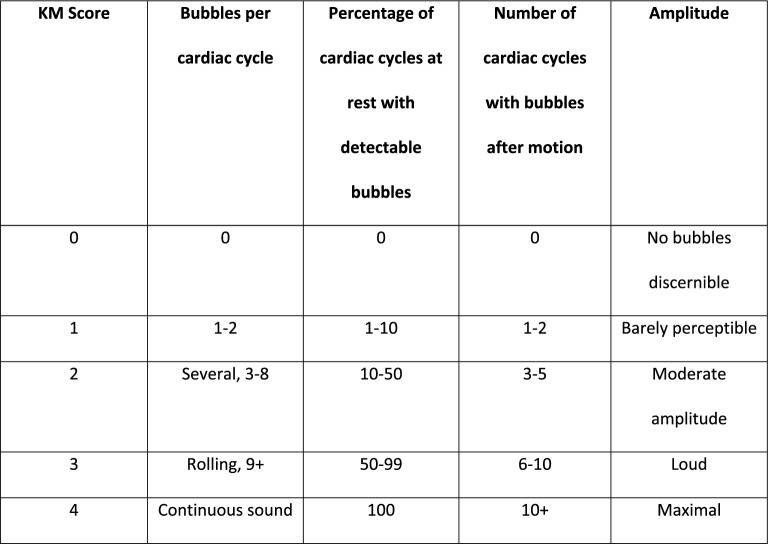
Kisman–Masurel (KM) precordial Doppler scoring system used to grade the quantity of venous gas via pulsed wave (PW) doppler and severity of decompression sickness

The work done by Karlsson et al. utilized a precordial continuous wave (CW) Doppler ultrasound to detect the presence of VGE in supine patients, simulating weightlessness, who were decompressed to an EVA suit’s pressure of 386 hPa for 6 h. [16] The quantification and diagnosis of VGE was carried out using the Kisman–Masurel (KM) precordial Doppler scoring system, terrestrially recognized to be one of the first classification methods in the field of decompression sickness research [17]. The KM scoring system characterizes the presence of acoustic bubble echoes within the cardiac cycle on CW doppler, ranging from an absence of bubbles to continuous, high-intensity bubble-echoes (Fig. 3) [18]. VGE was only detected by doppler ultrasound in two of the twenty patients, indicating that the simulated weightlessness may have decreased the incidence of VGE and DCS [16]. Nevertheless, the study established the use of precordial Doppler ultrasound for detecting VGE in patients in simulated weightlessness, but more research is needed in microgravity environments and to evaluate whether precordial ultrasound still can be a viable imaging location for detecting presence of VGE.

### Deep venous thrombosis

**Fig. 4:**
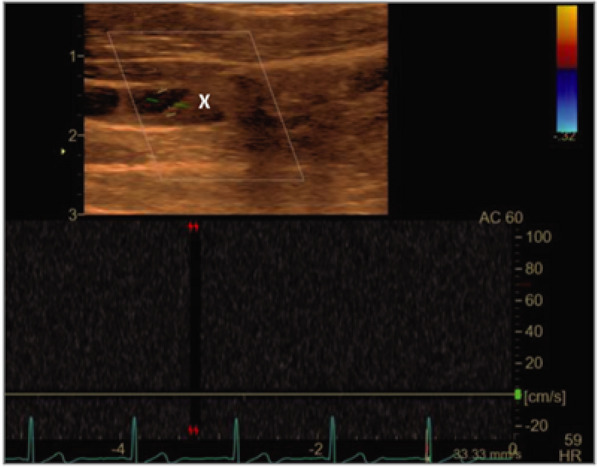
Internal jugular vein longitudinal ultrasonographic scan showing a thrombus (labeled X), and no doppler measured flow in the vessel [19]

Due to the headward fluid shift in microgravity, superior veins such as the internal jugular vein (IJV) become engorged with fluid and have been shown to have decreased or retrograde flow [19]. It is thought that the decreased flow and nondominant anatomical location of the left IJV can lead to increased risk of deep venous thrombosis (DVT) during spaceflight [19]. In a study conducted by Marshall-Goebel et al., ultrasound was used to diagnose one crewmember suspected to have an occlusive left IJV thrombus while on the ISS and was promptly treated with anticoagulation for the remaining duration of the flight (Fig. 4) [19]. The crewmember’s IJV was subsequently surveilled with ultrasound images at 7–21-day intervals, showing progressive reduction of the thrombus [19, 20]. Marshall-Goebel et al.’s study was the first of its kind using ultrasound to diagnose an acute DVT in space and proved to be effective in both obtaining high quality images for experienced sonographers on the ground to make the diagnosis as well as continued surveillance of the DVT’s response to treatment. More research is needed to further explore DVTs in the internal jugular and other superior veins.

### Pneumothorax and hemothorax

**Fig. 5:**
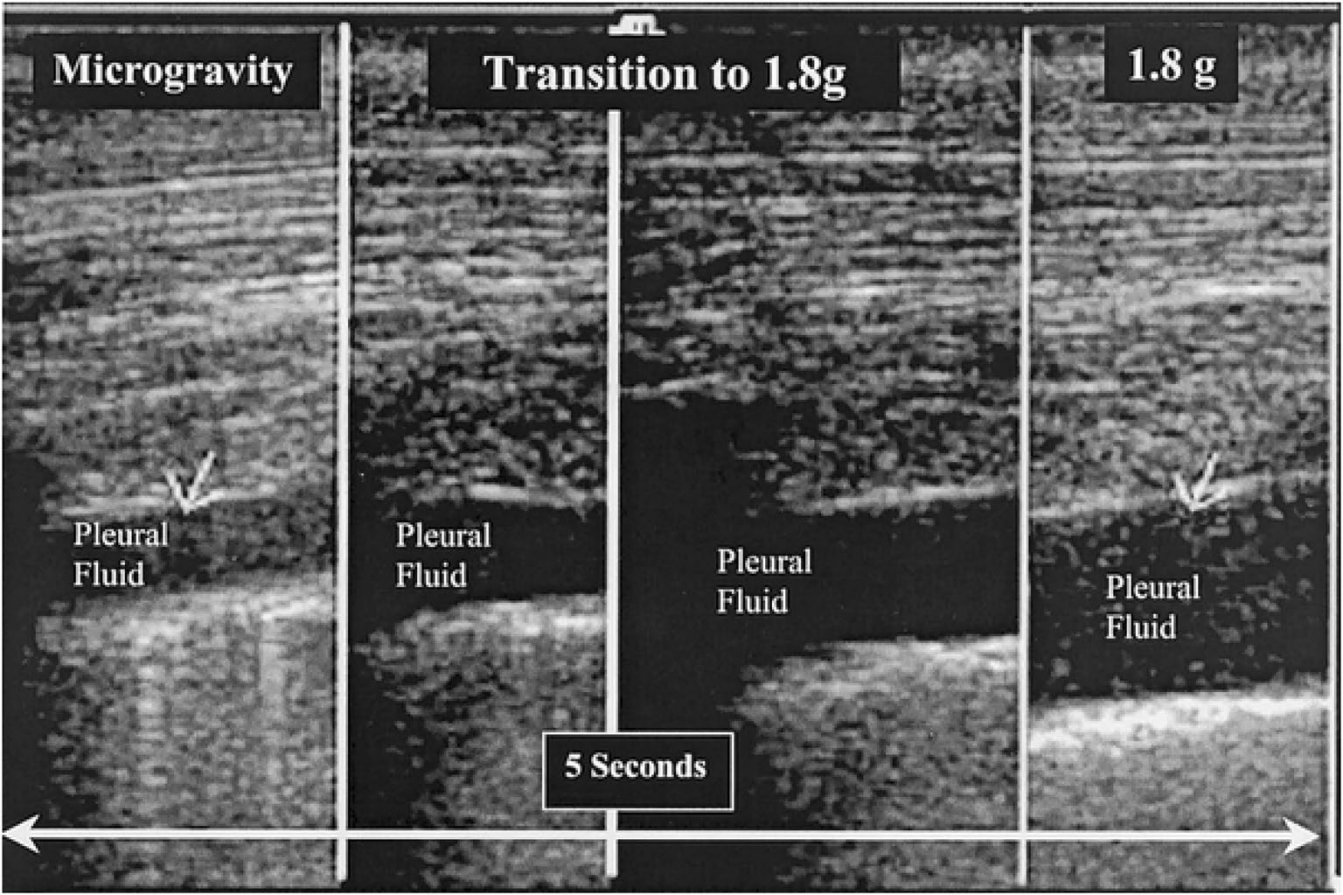
Ultrasound detected induced 100 mL hemothorax in the posterior thoracic window in and after microgravity [21]

One of the key components of a comprehensive trauma evaluation is excluding the presence of significant pneumothorax and hemothorax that could potentially compromise respiratory and hemodynamic stability. Terrestrially, the presence of “lung sliding” on ultrasound, which represents the pleura sliding across from each other, is a good indicator that a significant pneumothorax is not present. Hamilton et al. simulated a hemothorax and pneumothorax in a supine porcine model during parabolic flight by injecting aliquots of air or saline in the pleural cavity [21]. They found that during 0 g, the absence of lung sliding was detected in both anterior and posterior lung fields with relatively equal sensitivity once insufflating the pleural cavity with air. Lung sliding was then detected in all lung fields shortly after placing a chest tube to evacuate intrapleural air. Fluid was then injected through the chest tube and equally identified in both anterior and posterior lung windows at 50 ml (Fig. 5). These findings differed from the ground studies, where they found a progressive anterior-to-posterior disappearance of lung sliding as more air is insufflated. In addition, hemothorax was first detected in the posterior pleural cavity at 25 ml, whereas the anterior cavity could only detect hemothorax at 100 ml. This difference can be explained by the effect of gravity distributing fluid towards the dependent, posterior regions in the hemothorax model and air being displaced upwards by the gravity-dependent lung in the pneumothorax model. In contrast, fluid and air appears to be equally distributed in the pleural cavity in microgravity due to the absent force that displaces denser material posteriorly. The ability to effectively detect air and fluid in both anterior and posterior windows in microgravity makes ultrasound a promising modality for assessing thoracic trauma and preventing unnecessary tube thoracostomies and evacuations in spaceflight.

### Sinusitis

In microgravity, mucociliary drainage may be altered and could result in obstruction of the sinus passages. This, in combination with the dryer recirculated air in space vehicles, may lead to mucus stasis and increased predisposition to acute bacterial rhinosinusitis (ABRS). As a result of the headward fluid shift in space, astronauts frequently complain of sinus congestion and headaches during spaceflight. These common complaints make clinical diagnosis of ABRS, which is the standard of care on Earth, difficult. Ultrasound has been shown to be a viable tool in detecting maxillary sinusitis on Earth. In microgravity conditions, Benninger et al. developed a porcine sinusitis model, where they examined the maxillary sinus with ultrasound before and after injecting it with 1 mL of fluid during parabolic flight [22]. In normal gravity, the typical sonographic finding of an air–fluid interface was visualized. However, the air–fluid interface rapidly disassociated in microgravity as the fluid distributed along the walls of the sinus, which can be seen in the image in Benninger's work [22]. This is thought to be due to the surface tension of the fluid preferentially adhering to the walls of the sinus in the absence of gravity [22]. While the air–fluid interface was not detected on ultrasound in microgravity, the presence and thickness of a fluid layer along the walls of the sinus could be an adjunct sign in detecting sinusitis.

### Musculoskeletal trauma

It has been shown that exposure to microgravity leads to muscle atrophy and bone demineralization, predisposing astronauts to musculoskeletal injuries that can compromise a mission. One of the most common sites of injury is the rotator cuff, due to the constraints placed on the astronaut’s body when donning the extravehicular activity (EVA) suit [23]. The estimated incidence of shoulder injuries during EVA training exercises is 9.67 injuries per 100 astronauts over a 10 year period [24]. Fincke et al. reported the first use of musculoskeletal ultrasound on the ISS that evaluated a crewmember’s shoulder integrity. The images obtained by nonphysician crewmembers took a total of 15 min to acquire and were of high enough quality for an expert sonographer on Earth to rule out injury. [25]

**Fig. 6:**
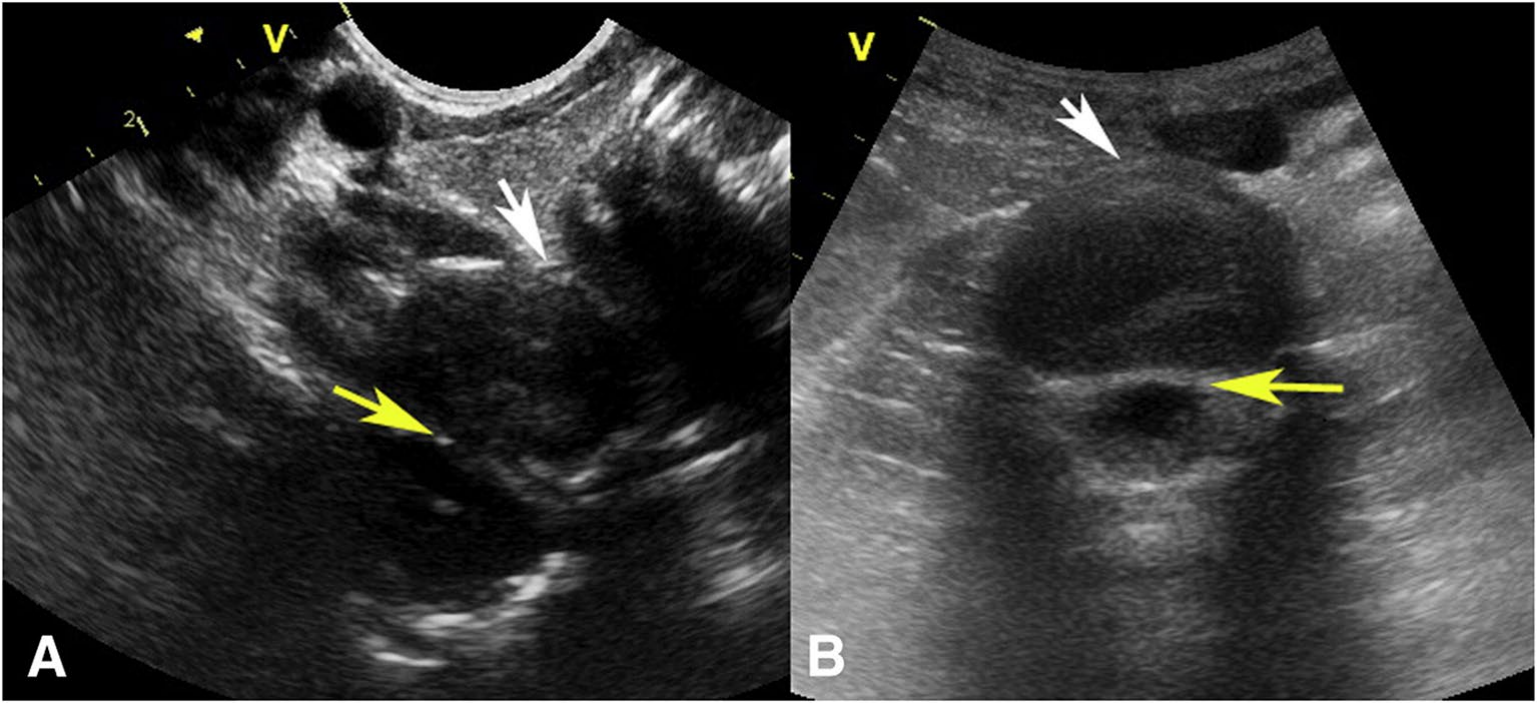
**A** Cervical spine transverse view through the cervical C5–6 intervertebral disk. **B** Lumbar spine transverse view through L3–4 intervertebral disk. The yellow arrow indicates the anterior longitudinal ligament. The white arrow indicates the posterior longitudinal ligament. An abnormality in this location would identify a bulge or herniation. A curvilinear probe was used [27]

Similar to an increased risk of shoulder injuries, the risk of herniated nucleus pulposus (HNP) in astronauts is 4.3 times higher than matched controls not involved in spaceflight, mostly occurring in the cervical spinal region [26]. Spinal injuries during spaceflight could be difficult to diagnose without imaging modalities, such as X-ray, CT, or MRI. Spinal ultrasound is rarely used in the clinical practice, making implementation of techniques to the ISS difficult to accomplish. However, Marshburn et al. demonstrated the first spinal ultrasound on the ISS and found that crewmembers with minimal training were able to obtain high quality spinal images of the cervical and lumbar spine (Fig. 6) [27]. A few years later, Garcia et al. implemented a formal spinal ultrasound protocol, where crewmembers on the ISS were able to visualize the cervical spine from C3 to T1 and the lumbar spine from L3 to S1 using ultrasound. The entire disk contour was able to be completely visualized on most exams which could allow terrestrial radiologists to identify tears or herniations. Moreover, among the crewmembers who were imaged, 14 new or progressive pathologic findings of the cervical and lumbar spine were found compared to preflight Imaging, such as disk desiccation, osteophytes, and qualitative changes in the intervertebral disk height and angle [28]. These studies have greatly advanced the capability of ultrasound on the ISS to serve as the primary diagnostic tool in evaluating for musculoskeletal injuries.

### Genitourinary emergencies

Due to the decreased loading of bone in microgravity, there is an increased release of calcium from the bone into the bloodstream [29]. As a result, a feared complication in space is nephrolithiasis, especially during extended space missions, where evacuations are infeasible. A salient example is a Russian cosmonaut aboard the Salyut, who experienced such severe pain from a urinary stone that there was almost an emergency evacuation [29]. Interestingly, a total of 11 US crewmembers had 14 urinary calculi following their missions averaging less than 2 weeks in duration [29]. The most common symptom of ureteral stones is intractable, fluctuating pain, called “renal colic,” and is usually located on the flank or abdominal quadrant of the side, where the stone is obstructing.

**Fig. 7:**
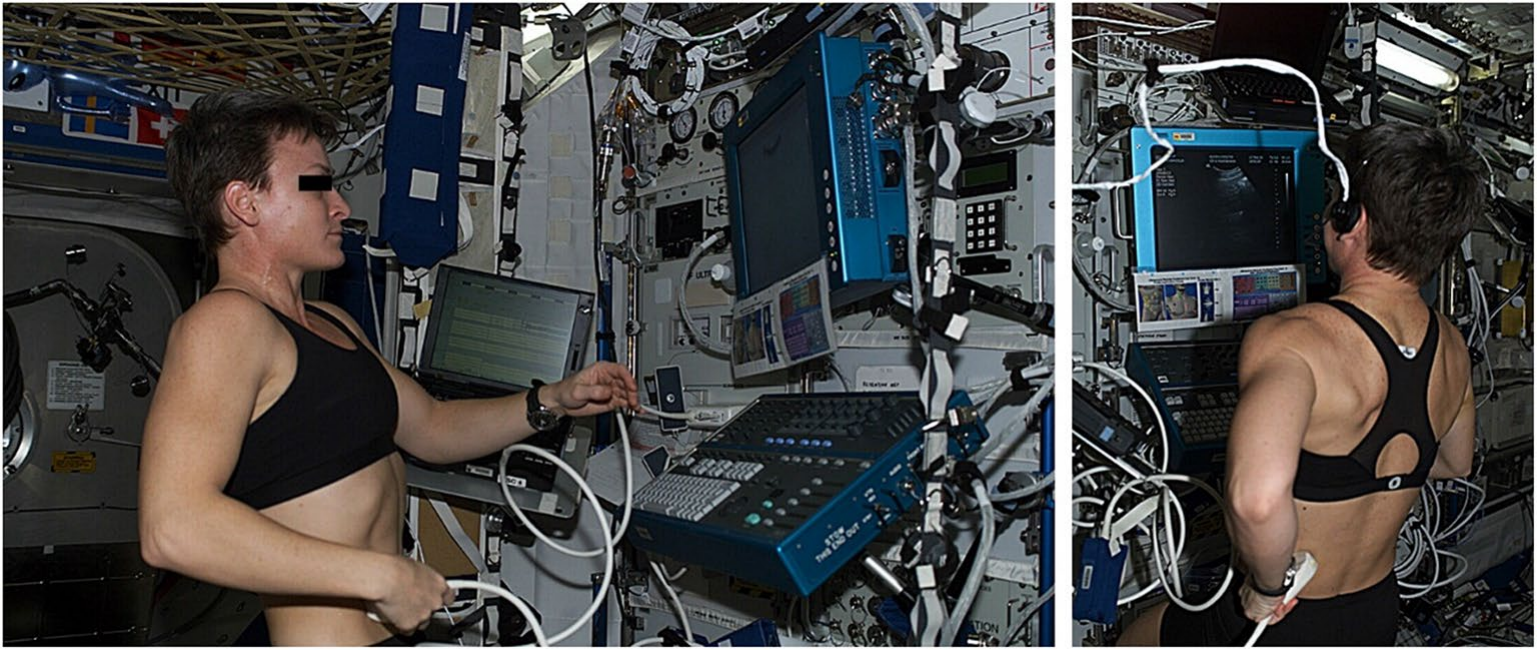
Crewmember aboard the ISS performing a guided renal ultrasound examination [31]

Without imaging modalities, such as X-ray or CT, flight surgeons are limited to ultrasound in distinguishing if a crewmember’s pain is due to renal colic or an intra-abdominal process. Terrestrially, using ultrasound to diagnose nephrolithiasis has been shown to have no significant difference in adverse events when compared to CT [30]. Jones et al. demonstrated that a remote-guided ultrasound of the genitourinary system (Fig. 7) was able to produce diagnostic-quality images of the kidney, ureters, and bladder. The images obtained by the crewmembers were able to be used to evaluate for the following: obstruction, masses, cysts, or perinephric processes in the kidney, bladder distension or lesions, percutaneous direction of suprapubic catheter for urinary retention treatment, drainage of urine into the bladder (termed “ureteral jets”), and the condition of the bladder wall. There were no obvious differences in anatomy due to microgravity that were appreciated on ultrasound and findings were comparable to images obtained on Earth [31].

**Fig. 8:**
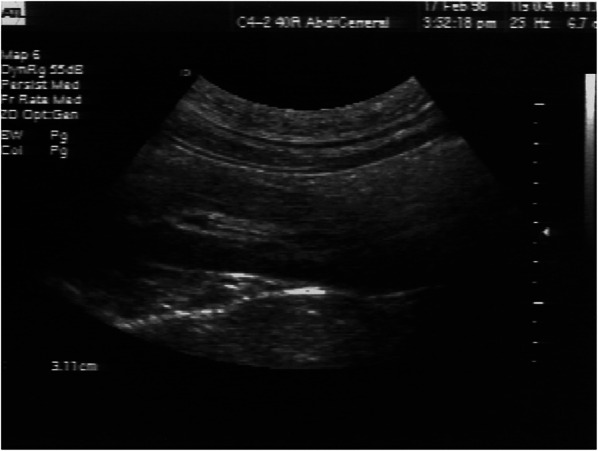
Sonographic view of the proximal ureter and renal pelvis with visualization of the echogenic guide wire and ureteral stent [32]

Typically, in the management of nephrolithiasis, small ureteral stones (usually under 10 mm) involve more conservative, non-invasive measures to allow the stone to pass, while larger, more obstructive stones require interventional procedures. Because lithotripsy machines are too heavy to be flown in a spacecraft, endoscopic ureteral stenting is the next best interventional option. However, ureteral stenting usually requires X-ray to confirm placement, and performing the procedure in the confines of a space vehicle while in microgravity increases the challenge. In a parabolic flight study conducted by Jones et al., endoscopic ureteral stenting was performed on a porcine model and ultrasound was used to follow the guide wire into the proximal ureter and renal pelvis (Fig. 8) [32]. The echogenic guidewire was appropriately visualized on ultrasound and the images obtained had no significant difference in quality as rated by experienced sonographers compared to the imaged obtained in the same model on Earth. The surgical technique was also feasible in microgravity, however, maintaining steady positioning of the operators around the patient and the management of floating irrigation fluids which could risk contaminating other crewmembers proved to be challenging and required sufficient planning [32]. In addition, if the stone is impacted into the ureteral wall or the anatomy of the ureter is tortuous, ultrasound may be inferior to fluoroscopy in being able to follow the placement of the guidewire. [32]

In another study, a porcine model was used to simulate urinary obstruction—a condition which could result from urolithiasis, especially in individuals who have a predisposing urethral impingement, such as strictures or benign prostatic hyperplasia [33]. Ultrasound successfully allowed for bladder visualization and guidance of a suprapubic catheter for drainage. The study also explored the detection of possible bladder rupture and subsequent urinary extravasation by introducing fluid into the pelvic space. On a porcine model, extravasated fluid was able to be detected at a threshold of 250 cc via a suprapubic transverse view [33].

### Ocular emergencies

Acute ocular pathology during spaceflight is not uncommon. A systematic review found that the most common acute ocular condition during NASA missions was corneal abrasion; however, chemical burns, eye debris, and ocular infections also occurred [34]. While these conditions are usually diagnosed clinically, astronauts may still experience an acute ocular emergency, such as serious trauma or retinal detachment requiring sonographic evaluation.

In a study conducted by Chiao et al., ISS crewmembers successfully conducted ocular ultrasound with minimal training in ultrasound via remote guidance from a sonographic expert. The iris, pupil, pupillary response, as well as the anterior and posterior segments of the globe were obtained with high quality and minimal differences to terrestrial images [7]. These findings reveal that ocular ultrasound in microgravity can be done by a minimally trained crewmember with remote expert guidance and detailed cue cards [7]. While in-flight ocular ultrasound has the potential to diagnose acute ocular pathology, such as globe disruption, lens dislocation, ocular foreign body, retinal/choroidal detachment, or retinal artery occlusion, future research is needed to determine if these pathologies present differently in microgravity.

## Conclusion

As space exploration advances, the need for effective medical diagnostics in microgravity becomes increasingly evident. This review has detailed the potential of ultrasound in diagnosing and managing a range of acute conditions in space, from abdominal trauma to ocular and musculoskeletal issues to procedural guidance. However, as we push further into space, there's a clear gap in our understanding of certain acute pathologies. Specifically, we lack comprehensive data on gastrointestinal emergencies related to the small bowel, biliary tract, pancreas, and appendix; cardiac issues, such as cardiac tamponade and myocardial infarction; vascular problems, such as dissections and pulmonary embolism; hemorrhages into the retroperitoneum; and reproductive emergencies, including ectopic pregnancy. As our reach into the cosmos expands, it's essential that our medical knowledge keeps pace. This ensures that as we undertake longer and more complex missions, we're fully prepared to address the unique medical challenges that arise, ensuring astronaut health and mission success.

### Supplementary Information

**Additional file 1.**  Supplementary file includes detailed search strategy used with specific search indexing strategy.

## Data Availability

Not applicable.
