# Correction: Utility of ultrasound in managing acute medical conditions in space: a scoping review

**DOI:** 10.1186/s13089-023-00353-2

**Published:** 2024-01-11

**Authors:** Parsa Asachi, Ghadi Ghanem, Jason Burton, Haig Aintablian, Alan Chiem

**Affiliations:** 1 David Geffen School of Medicine at UCLA, 855 Tiverton Dr, Los Angeles, CA 90024 USA; 2 University of California, Los Angeles Library, Los Angeles, CA USA; 3 Department of Emergency Medicine, David Geffen School of Medicine UCLA, Los Angeles, CA USA; 4 Department of Emergency Medicine, David Geffen School of Medicine UCLA, Olive View UCLA Medical Center, Los Angeles, CA USA

**Correction: The Ultrasound Journal (2023) 15:47** 10.1186/s13089-023-00349-y

After publication of this article [1], the authors reported that the given and family names of all authors were interchanged.

The original article [1] has been corrected.
