# Supplementary material for: Utility of ultrasound in managing acute medical conditions in space: a scoping review
Source: Ultrasound J. 2023 Dec 12;15:47. doi: 10.1186/s13089-023-00349-y (PMC10716092; doi:10.1186/s13089-023-00349-y)
Supplement: Supplementary file 1 — Additional file 1. Supplementary file includes detailed search strategy used with specific search indexing strategy. [file 13089_2023_349_MOESM1_ESM.docx]

# Supplement

Detailed search strategy:

Searches run February 25, 2023

PubMed: 421

("Ultrasonography"[Mesh] OR ultrasound OR Echotomography OR Sonography OR Ultrasonograph* OR Echography OR Echocardiography OR Echoencephalography OR Endosonograph* OR “Acoustic Microscop*” OR “Focused Assessment with Sonography for Trauma”) AND ("Weightlessness"[Mesh] OR “Zero Gravity” OR Microgravity)

s

Embase:222

('echography'/exp OR 'ultrasound'/exp OR 'echocardiography'/exp OR 'echotomography'/exp OR 'echoencephalography'/exp OR 'acoustic microscopy'/exp OR 'focused assessment with sonography for trauma'/exp OR sonography:ti,ab,kw) AND ('weightlessness'/exp OR 'microgravity'/exp OR ‘Zero Gravity’:ti,ab,kw)

Web of Science: 273

(Ultrasonograph* OR ultrasound OR Echotomography OR Sonography OR Echography OR Echocardiography OR Echoencephalography OR Endosonograph* OR “Acoustic Microscop*” OR “Focused Assessment with Sonography for Trauma”) AND ("Weightlessness" OR “Zero Gravity” OR Microgravity)

Total: 916

Deduplicated: 626
